# Sex differences in social modulation of learning in rats

**DOI:** 10.1038/srep18114

**Published:** 2015-12-14

**Authors:** Marta Mikosz, Aleksandra Nowak, Tomasz Werka, Ewelina Knapska

**Affiliations:** 1 Department of Neurophysiology, Nencki Institute of Experimental Biology PAS, 02-093, Warsaw, Poland

## Abstract

In its simplest form, empathy can be characterized as the capacity to share the emotional experiences among individuals, a phenomenon known as emotional contagion. Recent research shows that emotional contagion and its adaptive role can be studied in rodents. However, it is not known whether sex differences observed in human empathy extend to its more primitive forms. In the present study, we used a rat model of emotional contagion to compare the behavioral consequences of social transfer of information about threat, and the subsequent neural activation patterns in male and female rats. We found that: (1) males and females display a similar behavioral pattern during the interaction with either a fear-conditioned or a control rat; (2) interaction with a fear-conditioned conspecific positively modulates two-way avoidance learning in male and diestral female rats but not in estral females; and (3) such interaction results in increased c-Fos expression in the central and lateral nuclei of the amygdala and the prelimbic and infralimbic cortex in males, whereas in females no such changes were observed. Collectively, our results point to the occurrence of sex and estrus cycle phase differences in susceptibility to emotional contagion and underlying neuronal activation in rodents.

According to the perception-action model of empathy proposed by Preston and de Waal^1^, perception of the object’s emotional state automatically and unconsciously activates representations of such states in the subject as well as produces appropriate somatic responses. Viewed in this way, empathy encompasses phenomena, such as emotional contagion, which have important evolutionary consequences as they increase the probability of avoiding danger^2^. Broadening of the definition of empathy enabled researchers to study this phenomenon with reference to its evolutionary underpinnings, which seem to lie far beyond primates. The results of a number of recent studies using rodent models of empathy seem to confirm this notion (see ref. 3 for review).

Human research points to the existence of sex differences in empathy^4^,^5^,^6^. Research using rodent models was, with few exceptions^7^,^8^,^9^,^10^, performed exclusively in males. Even if both sexes were used, no subsequent comparison of male and female data was provided. Moreover, the accounting for the estrus cycle in studies using females is sparse, although the literature suggests that empathic abilities vary across menstrual cycle in women^11^,^12^,^13^,^14^.

In order to verify whether sex differences in empathy exist in rodents, we have employed a rat model of emotional contagion designed in our laboratory^15^,^16^. In this model, one animal from a pair that has been housed together (the “demonstrator”) undergoes fear conditioning. Subsequently, that animal is returned to its home cage and can interact with its cage mate (the “observer”) that is otherwise naïve. According to our previous results, social transfer of emotional information occurs during that interaction and is sufficient to induce plastic changes in the amygdala, as assessed by c-Fos expression analysis and modified learning in the male observers^15^,^16^.

In the current study, we carried out four experiments designed to: (1) compare male and ovariectomized female rats as demonstrators (Experiment 1); compare behavior of male and female observers during an interaction with a shocked demonstrator (Experiment 2); examine effects of the interaction on acquisition of active avoidance in observers (Experiment 3) and examine c-Fos expression patterns in brain structures involved in processing of socially transferred emotional information, the central and lateral nuclei of the amygdala^15^, prefrontal cortex^17^ and insular cortex^18^ (Experiment 4). To investigate influence of the estrus cycle phase on emotional contagion in rats, we have performed the experiments in females in estral and diestral phases of the estrus cycle.

## Results

### Experiment 1: Male and female demonstrators show similar fear memory following contextual fear conditioning and have similar pain thresholds

**Figure 1:**
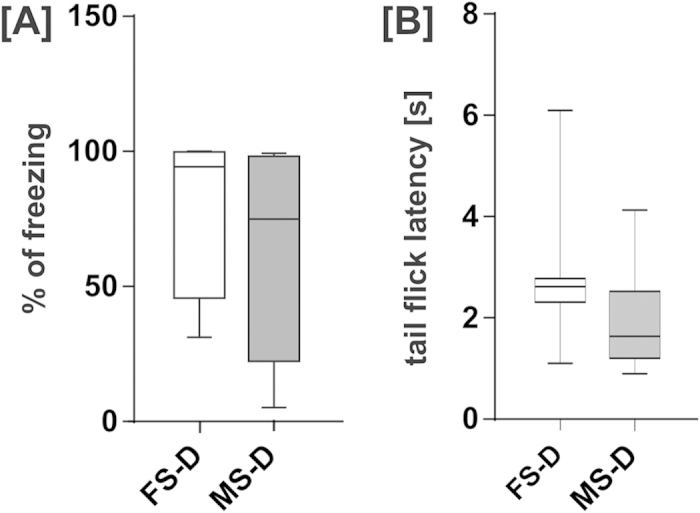
Male (MS-D) and female (FS-D) demonstrators show similar contextual fear memory and pain thresholds. (**A**) Freezing response during contextual fear memory recall did not differ between male and ovariectomized female demonstrators. (**B**) Tail-flick test did not reveal any significant differences between responses to an acute thermal stimulus. Median value, interquartile range and range are presented.

In order to examine whether male and ovariectomized female demonstrators comprise a comparable source of emotional information for the observers, demonstrators’ fear memory, which is related to the level of emotional arousal during fear conditioning, and pain threshold were tested. We analyzed freezing response following contextual fear conditioning using a protocol which was previously employed to produce emotional arousal in demonstrator rats^16^. Two groups of animals were compared, male (MS-D) and ovariectomized female rats (FS-D), see Fig. 1A. For a detailed description of the experimental groups see Table 1. No statistically significant difference between male and ovariectomized female demonstrators was observed (Mann-Whitney test, U = 21, p = 0.27). Then, we assessed pain thresholds in the same group of male and ovariectomized female rat demonstrators using the tail-flick test (Fig. 1B). Tail-flick latencies of male and ovariectomized female demonstrators did not differ (Mann-Whitney test, U = 16, p = 0.105). The results indicate that ovariectomized females and males experience similar levels of fear during training, which translates to comparable fear memory.

### Experiment 2: Male and female observers behave similarly during social interaction with a fear-conditioned demonstrator

Since it has been shown that visual and olfactory cues play important role in social modulation of learning^19^, we compared behaviors of male and female rats during social interaction with emotionally aroused partner. We observed that both male and female demonstrators and observers engage in social exploratory behaviors rather than aggressive encounters. It is consistent with our previous observations for interaction of male rats^16^. Moreover, we analyzed the number and/or duration of the following behaviors in males, and estral and diestral females: of non-social exploratory behaviors: (1) rearing, (2) digging, (3) short self-grooming (head area) (4) long self-grooming (whole body); of social exploratory behaviors - (5) sniffing partner in the anogenital area, (6) sniffing partner in the head area and (7) allogrooming (grooming of the partner). Kruskal-Wallis test revealed significant between-group differences for duration of rearings (H(5,48) = 11.326, p < 0.05), as well as number and collective duration of head sniffing episodes [(H5,48) = 15.089, p < 0.05 and H(5,48) = 11.881, p < 0.05, respectively]. Results and statistically significant pairwise comparisons using Mann-Whitney test are summarized in Table 2. The results indicate that during the interaction, the observers were involved mainly in social exploratory behaviors; these behaviors were directed at their partners, and aimed at gaining information. No significant differences between male, and estral and diestral female observers have been observed.

### Experiment 3: Interaction with a recently fear-conditioned demonstrator sex-dependently influences acquisition and retention of two-way avoidance response

We have previously shown that interaction with an animal that underwent fear conditioning, and is thus emotionally aroused, enhances shock-motivated active avoidance learning in males^16^. In order to determine whether sex and cycle phase in female rats influence the social modulation of active avoidance learning, we compared different parameters of learning in male (M), diestral female (D) and estral female (E) observers paired with either a fear-conditioned demonstrator (shocked, S) or an animal which was merely exposed to the experimental cage (non-shocked, NS). We also examined retention of the two-way avoidance response in observers previously subjected to an interaction with a shocked or a non-shocked demonstrator.

**Figure 2:**
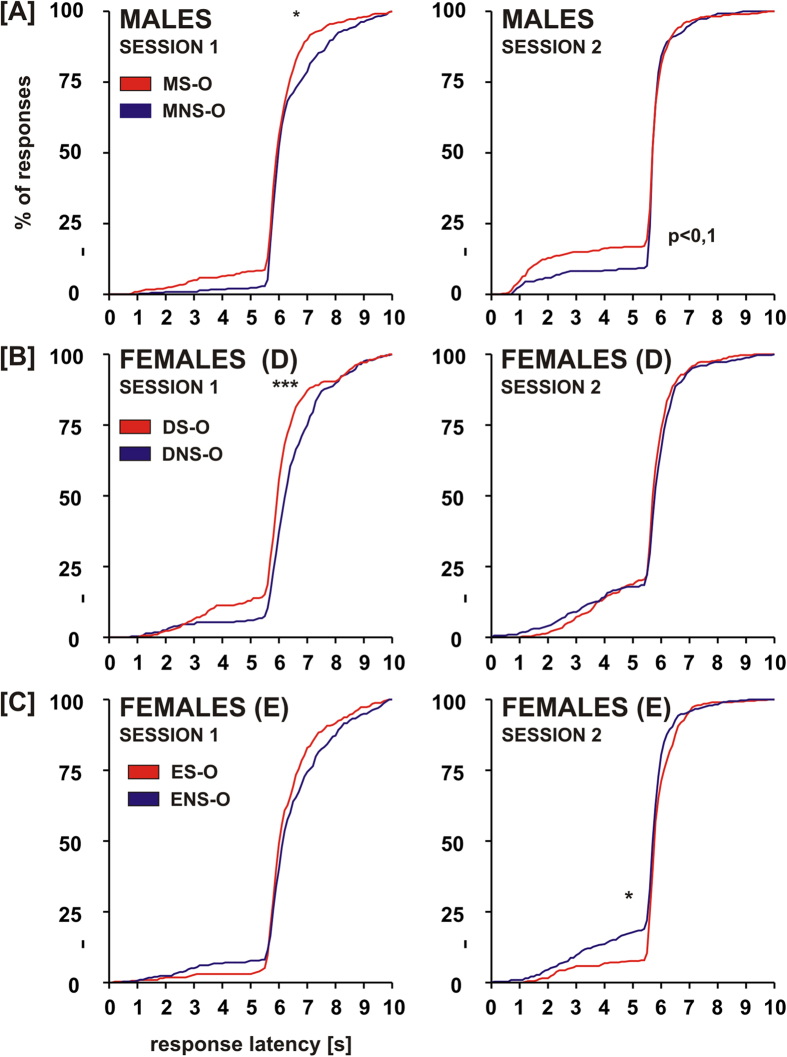
Interaction with a recently fear-conditioned animal improves acquisition of two-way avoidance response in males and diestral females. Cumulated distribution of latency of avoidance (shorter than 5 sec) and escape responses (longer than 5 sec) in two training sessions is shown. Levels of significance (Kolomogorov-Smirnov two-sample test): *p < 0.05, **p < 0.01, ***p < 0.001.

Two-way avoidance acquisition and retention were analyzed using instrumental response latencies and number of avoidance responses. The cumulative latency plots were compared using the Kolomogorov-Smirnov two-sample test (Fig. 2). Comparison of the S groups and their NS counterparts revealed that in the 1^st^ training session (acquisition) males from the MS-O group had significantly shorter latencies of escape responses (D_max_(6.9 s) = 0.119, p < 0.025) than males from the MNS-O group. In the 2^nd^ training session (retention) this difference was less apparent and did not reach significance level (D_max_(5.5 s) = 0.093, p < 0.1). Females from the DS-O also performed faster instrumental responses than their DNS-O counterparts in the 1^st^ training session (D_max_(6.1 s) = 0.193, p < 0.001). In contrast to males and diestral females, no differences between ES-O and ENS-O groups were found in the 1^st^ training session. In the 2^nd^ session females from the ES-O performed instrumental responses with longer latencies than the ENS-O group (D_max_(5.5 s) = 0.116, p < 0.025).

Comparison within the S and NS groups revealed that in the 1^st^ session males from the MS-O group performed faster instrumental responses than the ES-O group (D_max_(6.4 s) = 0.107, p < 0.05) and males from the MNS-O group had shorter response latencies than the DNS-O and ENS-O groups (D_max_(6.1 s) = 0.17, p < 0.001 and D_max_(6.0 s) = 0.121, p < 0.025). In the 2^nd^ session of the two-way avoidance training, males from the MS-O group showed shorter latencies of avoidance responses than the DS-O group (D_max_(5.9 s) = 0.182, p < 0.025), as well as shorter latencies of both avoidance and escape responses than the ES-O group (D_max_(5.5 s) = 0.119, p < 0.01).

The number of avoidance responses was analyzed in blocks of 10 trials. Kruskal-Wallis test revealed the presence of statistically significant differences between groups (H(59,450) = 155.64, p < 0.001). In the S groups, the DS-O rats performed significantly better than the ES-O group in the 4^th^ and 5^th^ block of the 1^st^ session and in the 4^th^ block of the 2^nd^ session (Mann-Whitney test, U = 4, p < 0.005; U = 10, p < 0.05 and U = 10, p < 0.05, respectively). We did not observe any significant differences between sexes and estrus cycle phases in the NS groups. Pairwise comparisons between the S and NS groups showed that the DS-O group performed significantly better than the DNS-O group in the 4^th^ block of the 1^st^ training session (U = 6, p < 0.005). The ES-O group performed better than the ENS-O group in the 4^th^ block of the 1^st^ session (U = 12, p < 0.05), however it performed worse in the 2^nd^ and 4^th^ block of the 2^nd^ session (U = 8, p < 0.05 and U = 8.5, p < 0.05, respectively).

The results show that interaction with a fear-conditioned demonstrator facilitates immediate acquisition of two-way avoidance in male and diestral female observers, and have no effect on avoidance acquisition in estral females. Moreover, in estral females such interaction worsens two-way avoidance retention.

Since we have previously found that interaction with a fear-conditioned demonstrator increases number of intertrial responses (ITRs) – crossings between shuttle box compartments during intertrial intervals when neither conditioned stimulus (CS) nor unconditioned stimulus (US) is present^16^ – we have compared this parameter in male and female observers. ITRs were most abundant in the first block of the first training session. Pairwise comparisons between groups within the same block of trials did not reveal any statistically significant differences (Kruskal-Wallis test, H(59,450) = 155.64, p < 0.001) followed by Mann-Whitney test).

### Experiment 4: Interaction with a recently fear-conditioned demonstrator results in activation of central and lateral amygdala and prefrontal cortex only in male rats

As shown previously by our group, interaction with a fear-conditioned conspecific strongly activates amygdalar nuclei in male rats. The pattern of c-Fos activation in the observer’s amygdala generally paralleled that of the shocked demonstrators, however c-Fos expression in the central nucleus of the amygdala was elevated specifically in the observers^15^. In order to broaden our knowledge on the neuronal circuit processing social emotional information and determine whether an interaction with a feared conspecific activates the same neuronal circuits in the brain of male and female rats, we performed a c-Fos expression analysis in the lateral and central amygdala, but also in the prefrontal cortex (separately for the prelimbic and infralimbic parts) and in the insular cortex. The central amygdala was divided into the lateral and medial subdivisions, which differ functionally^20^.

### Amygdala

We observed increased level of activation in the lateral and central amygdala (both lateral and medial parts) in the male observers paired with emotionally aroused demonstrators. Such increase was, however, observed neither in the estral nor in the diestral female observers.

**Figure 3:**
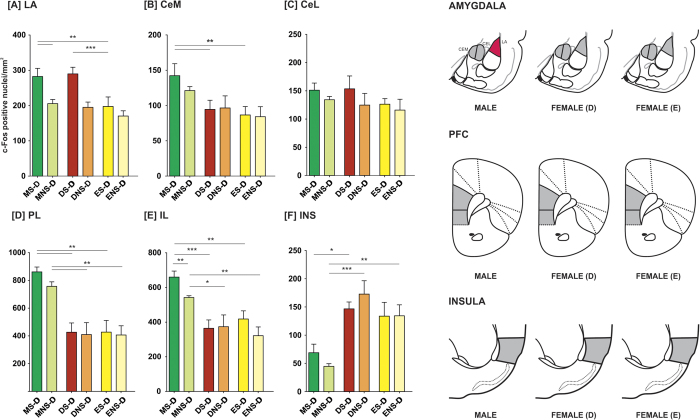
Fear conditioning followed by an interaction with a naïve conspecific induces c-Fos expression in the lateral nucleus of the amygdala. Mean number of c-Fos-immunopositive nuclei ± SEM per mm^2^ is presented. CeM – medial subdivision of the central amygdala, CeL – lateral subdivision of the central amygdala, LA – lateral nucleus of the amygdala, INS – insular cortex, PL – prelimbic cortex, IL – infralimbic cortex. In the right panel, activation maps are presented. Levels of significance (LSD test): ***p < 0.001 (red), S-O vs NS-O groups.

**Figure 4:**
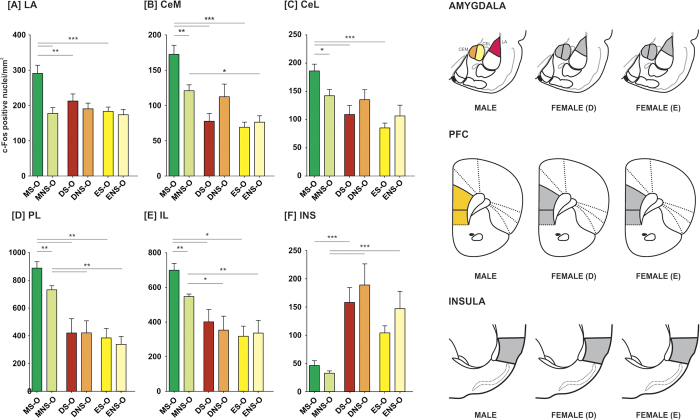
Interaction with a recently fear-conditioned conspecific induces c-Fos expression in the central and lateral nuclei of the amygdala and in the prefrontal cortex in males but not in females. Mean number of c-Fos-immunopositive nuclei ± SEM per mm^2^ is presented. CeM – medial subdivision of the central amygdala, CeL – lateral subdivision of the central amygdala, LA – lateral nucleus of the amygdala, INS – insular cortex, PL – prelimbic cortex, IL – infralimbic cortex. In the right panel, activation maps are presented. Levels of significance (LSD test): *p < 0.05 (yellow), **p < 0.01 (orange), ***p < 0.001 (red), S-O vs NS-O groups.

In the lateral nucleus of the amygdala (LA), factorial ANOVA (sex/estrus cycle phase x demonstrator/observer x demonstrator treatment) revealed the significance of sex [F(2,92) = 10.785, p < 0.001], demonstrator’s treatment [F(1,93) = 30.028; p < 0.001] and an interaction of these two factors [F(2,92) = 4.457, p < 0.05]. Demonstrators from the MS-D and DS-D groups had higher densities of c-Fos-positive nuclei in the LA comparing to the ES-D group (p < 0.01 and p < 0.001, respectively; Fisher LSD test; Fig. 3A). In observers paired with shocked demonstrators, males had higher numbers of c-Fos positive nuclei than females from the DS-O and ES-O groups (p < 0.01 and p < 0.001, respectively; Fig. 4A). A comparison of observers paired with shocked and non-shocked demonstrators revealed an elevation in c-Fos expression in males from MS-O, as compared to MNS-O group (p < 0.001).

In the central amygdala, factorial ANOVA revealed statistical significance of sex and estrus cycle phase both in the medial (CeM) and the lateral (CeL) subdivisions [F(2,95) = 24.379, p < 0.001 and F(2,95) = 8.713, p < 0.001]. Moreover, significance of the interaction of sex/estrus cycle phase and emotional arousal of the demonstrator was found in the CeM [F(1,96) = 5.005, p < 0.01]. In observers subjected to an interaction with a fear-conditioned demonstrator, males had significantly higher c-Fos expression than diestral and estral females, both in the CeM and CeL (in all cases p < 0.001, Fig. 4B,C). There were no differences between estrus cycle phases in either subdivision. In observers that have interacted with demonstrators merely exposed to novel environment, males had higher c-Fos positive nuclei densities than estral but not diestral females in the CeM (p < 0.05, Fig. 4B). No differences were found in the CeL (Fig. 4C). A comparison of observers interacting with fear-conditioned demonstrators and their NS counterparts revealed that the MS-O group had significantly higher number of c-Fos positive nuclei in the CeM as well as CeL (p < 0.01 and p < 0.05, respectively). In demonstrators, the rats from the MS-D group had significantly higher numbers of c-Fos positive cell nuclei in the CeM than rats from both the DS-D and ES-D groups (p < 0.01 and p < 0.01, respectively, Fig. 3B). Neither fear-conditioned nor control demonstrators had different c-Fos positive nuclei densities in the CeL, Fig. 3C.

### Cortex

We observed increased level of activation in the prelimbic (PL) and infralimbic (IL) parts of the prefrontal cortex in male observers paired with emotionally aroused demonstrators. Such increase was, however, observed neither in the estral nor in the diestral female observers (Fig. 4D,E). Factorial ANOVA revealed significance of sex in both the PL and IL [F(2,94) = 48.976, p < 0.001 and F(2,94) = 29.788, p < 0.001], as well as significance of the demonstrator’s treatment in the IL [F(1,95) = 4.371, p < 0.05]. In the PL, all groups of males had higher c-Fos expression than all groups of females (in all cases p < 0.01; Fig. 3D and 4D). In the IL, males from the MS-D group had significantly higher density of c-Fos-immunopositive cell nuclei than females from both DS-D and ES-D groups (p<0.001 and p<0.01, respectively; Fig. 3E) and male demonstrators from the MNS-D group had higher c-Fos expression than the DNS-D and ENS-D groups (p < 0.05 and p < 0.01, respectively). In the IL of the observers, the MS-O group had higher c-Fos expression than the DS-O and ES-O groups (p < 0.05) and the MNS-O group had higher c-Fos expression than the DNS-O and ENS-O groups (p < 0.05 and p < 0.01, respectively; Fig. 4D). Both in the PL and IL, Fisher LSD test did not reveal any differences between the S-O and NS-O groups of any sex. However, because factorial ANOVA revealed the significance of sex, we have performed additional one-way ANOVAs within sex groups. We have identified statistically significant differences only in the group of males (p < 0.001). Fisher LSD test revealed a significant difference between the MS-O and MNS-O groups both in the PL and IL (p < 0.01), as well as a difference between MS-D and MNS-D groups in the IL (p<0.01).

In the insular cortex we observed significantly higher c-Fos expression in females as compared to males. However, the level of activation did not reflect experimental treatment. Factorial ANOVA revealed statistical significance of sex [F(2,92) = 30.362, p < 0.001]. In the S groups, the DS-D but not the ES-D had higher numbers of c-Fos-positive nuclei than males (p < 0.05; Fig. 3F). In the NS demonstrators, the DNS-D and ENS-D had higher amounts of c-Fos-positive nuclei than males (p < 0.001 and p < 0.01, respectively). In observers from the S group, DS-O but not ES-O females had higher c-Fos expression than males (p < 0.001; Fig. 4F). Observers from the MNS-O groups had significantly lower c-Fos expression than observers from the DNS-O and ENS-O groups (in both cases p<0.001).

## Discussion

The main findings of the study are the following: (1) Comparable fear memory following a fear conditioning protocol used to emotionally arouse demonstrator rats, as well as similar pain thresholds in the demonstrator rats, indicate that male and ovariectomized female demonstrators are equivalent as a source of emotional information. (2) Male, estral female and diestral female observers are engaged in social exploratory behaviors; all animals show a similar behavioral pattern during the interaction with both a fear-conditioned and a control demonstrator rat. (3) Interaction with a fear-conditioned demonstrator positively modulates two-way avoidance learning in male and diestral female observers, but only in male observers it improves memory retention. (4) In male observers, interaction with a fear-conditioned demonstrator results in elevated c-Fos expression in the central and lateral nuclei of the amygdala and in the prelimbic and infralimbic parts of the prefrontal cortex. Such activation is not observed in female observers in either estrus cycle phase studied.

Our model originally employed males as both demonstrators (sources of emotional information), and observers (receivers of that information). The current adaptation of that protocol designed to study intact, cycling female observers required the use of demonstrators of the same sex. In order to exclude the effects of the demonstrator’s estrus cycle on the observer’s behavior, we decided to employ ovariectomized females as demonstrators. A question arose whether male and ovariectomized female demonstrators are a comparable source of stimuli for the observers. We addressed this issue by comparing the animals’ fear memory after exposure to contextual fear conditioning protocol which has been used to emotionally arouse the demonstrators in our previous studies^15^,^16^. Freezing response, a measure of fear memory, is correlated with unconditioned stimulus intensity^21^. Therefore, we hypothesized that if the stimuli used during fear conditioning would have been perceived as more aversive by the animal, they would result in higher freezing response. However, in accordance with the literature, we found no differences in freezing between ovariectomized female and male demonstrators^22^,^23^,^24^. We also found no differences in pain reactivity. Due to a lack of substantial evidence for differences between male and ovariectomized female demonstrators, we employed them in our study as equivalent sources of emotional stimulation for the observers.

Our previous results show that a brief interaction with a shocked demonstrator is sufficient to influence the observer’s learning^16^. We hypothesized that if sex differences exist in the social transfer of emotional information, they might be a result of differences in the observers’ investigatory behavior during the interaction. We did not, however, find any differences between males and diestral as well as estral females. This may suggest that they follow a similar behavioral pattern to acquire information about the emotional state of the partner and to investigate the interaction scene.

All observers were engaged in social exploratory behaviors, i.e., sniffing of the head and anogenital area of the demonstrator. Sniffing behavior is probably directed at gathering olfactory information about the emotional state of the demonstrator. In males, the perianal zone and the whisker pad were shown to excrete alarm pheromones that modify behavior and autonomous responses of their recipients^25^. The anal gland is not sexually dimorphic^25^ and the release of this pheromone is testosterone independent^26^. On the other hand, the release of the whisker pad pheromone is testosterone dependent. However, Rottman and Snowdon (1972)^42^ found in mice that both males and females release pheromones inducing an avoidance response. Nonetheless, the alarm pheromones and the effects exerted by them during the interaction with a fear-conditioned partner need further studies. Intense sniffing behavior however, observed in female observers, suggests that the observers are attracted by olfactory stimuli excreted by the demonstrator rats.

Albeit the emotional state of the demonstrator does not influence the behavior of observers during the interaction, it exerts influence on the initial phase of two-way avoidance learning. We found that in the first training session, immediately following the interaction, males as well as diestral, but not estral, females paired with shocked demonstrators had shorter instrumental response latencies than their counterparts paired with demonstrators exposed to a novel environment. For males the results are consistent with our previous observations^16^. One possibility to account for the differences between estral female and diestral female and male observers is that the emotional stimuli originating from the demonstrators are less likely to attract the estral observer’s attention. Attention is mediated by the noradrenergic system^27^ and females in estrus were shown to have the lowest noradrenaline levels in the amygdala^28^. It is also plausible that estral females are less anxious, as in the wilderness they explore beyond their home range while actively seeking males^29^. This effect is also seen in laboratory rats, as estral females show greater exploration of the elevated plus maze^30^.

In contrast to our previous data, male observers paired with shocked demonstrators did not show significantly better performance in the second session, however the difference was close to the significance level of 0.05. The discrepancy may stem from an interaction of two factors. In the experiments described herein we used animals from the Long-Evans strain, whereas previously we used animals from the Wistar strain which were shown to be inferior avoidance learners^31^. We also used a shorter and lower-ceiling shuttle box than previously, which was found to negatively affect two-way avoidance learning^32^. In contrast to males, diestral females paired with shocked demonstrators did not show any tendencies towards improved performance in the retention session and estral females paired with shocked demonstrators showed significantly poorer performance in the retention session. The tendency towards facilitated two-way avoidance memory retention is likely to be an effect of memory enhancement by modulatory systems. Agents that are best known for their memory-facilitating effects are noradrenaline and glucocorticoids (for review see ref. 33). Noradrenaline is required for consolidation of two-way avoidance memory^34^,^35^ and noradrenaline level in the basolateral amygdala following two-way avoidance training is positively correlated with memory retention 24 h later^36^. We hypothesize that the interaction with a shocked conspecific is emotionally arousing and results with an increase in noradrenaline release from locus coeruleus projections in the amygdala^27^. That, in turn, facilitates memory consolidation following two-way avoidance training in males, but not in females. While the lack of differences seen in diestral femals paired with shocked demonstrators in the retention session could be explained by weaker noradrenergic modulation, the poorer performance of estral females is hard to explain and deserves further investigation.

In male rats interaction with a partner subjected to fear conditioning results in learning improvement which is accompanied by elevated c-Fos expression in the central and lateral nuclei of the amygdala and in the prelimbic and infralimbic parts of the prefrontal cortex. Such activation is not observed in females. The results in male observers are very consistent with our previous observations for the amygdala^15^. Additionally, we show activation of the prefrontal cortex. Both the amygdala and the prefrontal cortex are involved in two-way avoidance learning^37^. Thus, it seems possible that social transfer activates or modulates neuronal circuits subsequently recruited by learning and thus decreases threshold of activation required for plastic changes to occur. In contrast, in diestral females we have observed modulation of instrumental responses performance during initial avoidance acquisition, with no concomitant effects on memory retention. The behavioral effects were accompanied neither by changes in activation of the amygdala nor in the prefrontal cortex, which is puzzling. One may be tempted to think that this result could be explained by the fact that the number of c-Fos positive cell nuclei in the examined structures was correlated with the amount of investigatory behaviors; however this explanation is not supported by the analysis of behavior during social interaction in females. Another factor that could have interfered with our results was the variability of estradiol levels. Estrogen levels in discrete brain structures vary between males and females^38^. Moreover, Weisz and Rosales^39^ have shown that the *c-fos* gene contains an estrogen receptor binding site upstream from the transcription initiation site and that estradiol stimulates c-Fos expression in estrogen-responsive cells. However, it seems unlikely that the impact of estrogen levels was sufficient to overshadow an activation, as other researchers have successfully employed c-Fos to investigate sex differences in neuronal circuit activation^40^,^41^.

Therefore, there are a few possible explanations of this puzzle. One may envision that in females, in the investigated brain structures the activity measured by c-Fos expression was weaker and fell below detection level or that other brain structures, not studied here, were activated. The effect may also stem from evolutionary roots (see below): that the presented stimuli have different cognitive values for males and females, and that females are more sensitive to other cues or that different neuronal circuits are activated in females and in males, which is very difficult to study with such global activity measure as c-Fos expression. Such difference may also result from sex differences in the function of neuronal circuits of the amygdala and prefrontal cortex, as shown by Gruene and colleagues^42^ in case of fear conditioning and extinction. We conclude that the observed lack of a clear relationship between behavioral interactions and the level of c-Fos activity opens up an interesting research direction.

Human studies point at sex differences in empathy and discrete differences in neuronal correlates of empathic processes between men and women^6^. Social structure of human and rat families is however hardly comparable. Laboratory rat strains are derived from Norway rats, which are social animals, but not cooperative and exhibit a limited set of social behaviors in the wild^44^. The behavior of male and female individuals is noticeably different; males are territorial and engage in aggressive encounters with other males, forming dominance structures. Territoriality in females, on the other hand, is limited to pregnancy and offspring care, dominance is less pronounced and aggression is lower. Moreover, females have smaller home ranges and explore beyond them only while actively attracting males in estrus^44^,^45^. The present, as well as our previous data^16^ suggest that fear is transferred between conspecifics through social interaction and promotes defensive responses in male observers that interact with a recently shocked demonstrator. It has also been shown that such responses may occur to a similar extent in male and female rats^7^,^46^. Thus, socially transferred fear can be an adaptation that promotes defensive behavior to potentially dangerous situations in the environment. Interestingly, in our experiments such effects in females are weaker or even do not exist (in estrus phase of the cycle), suggesting different behavioral and neuronal mechanisms of emotional contagion in females, presumably also differences in sensistivity to various social stimuli, that can be explained by different requirements of their natural environment.

## Material and Methods

### Experiment 1a: Analysis of fear memory in demonstrator rats

#### Subjects

Subjects were 16 naïve male and 16 naïve female Long-Evans rats, weighing respectively approx. 250-300 g and 160–210 g at the beginning of the experiment. Animals were supplied by the Nencki Institute Animal House. Upon arrival, males were randomly paired. Intact females were paired with ovariectomized females (for details see below). Rats were housed in pairs in standard housing cages (43.0 × 25.0 × 18.5 cm) under a 12/12 light–dark cycle, with food and water provided ad libitum. All animals were habituated to experimenter’s hand, transport, and separation for 14 days. The experiment was carried out in accordance with the Polish Act on Animal Welfare. All experimental procedures were approved by the First Warsaw Ethical Committee on Animal Research.

#### Ovariectomy

Half of the female rats underwent ovariectomy. Animals were anaesthetized with a mix of ketamine and xylazine (80 mg/kg and 10 mg/kg, respectively; Ketamina and Sedazin, Biowet, Poland). The dorsal surgical area was shaved and disinfected with ethyl alcohol. Bilateral skin and abdominal muscle wall incisions were made to access the peritoneal cavity. Ovaries and oviducts were exteriorized and ligatures were placed at the oviducts. Ovaries and parts of the oviduct were cut off above the ligature. The muscle wall and skin were sutured using absorbable and non-absorbable ligatures, respectively. Antibiotic ointment was applied on the wound and subcutaneous injections of tolfenamic acid (Tolfedine, Vetoquinol, France) were given and animals were placed on a heating pad for 1 h post-surgery. Thereafter the animals were single housed for one week for recovery.

#### Apparatus

A set of four fear conditioning chambers (Med Associates) was used for training and testing. Chambers were situated in sound-attenuating chests located in an isolated room. Ventilation fans built into the chests provided background noise (65 dB). Rear wall, ceiling, and hinged front door of all chambers were made of plexiglass and two side walls of each chamber were made of aluminum. The floor of each chamber consisted of 36 stainless-steel rods (3.2 mm diameter) spaced 8.0 mm apart. Floors were wired to a shock source and solid-state grid scrambler (Med Associates) for delivery of the footshock (unconditioned stimulus, US). US intensity was calibrated at the beginning of every experimental session. Illumination was provided by a built-in white and NIR light sources. A built-in video camera with a NIR filter was used to record the behavior of the animals. VideoFreeze software (Med Associates) was used to assess freezing behavior in the video recordings. Between tests, the chambers were cleaned with a detergent solution.

#### Procedure

The procedure was carried out as described previously^15^. Pairs of rats were randomly assigned to S (shocked) and NS (non-shocked) groups. In males (M), one animal was randomly designated as a demonstrator (D) and the other as an observer (O). In females (F), the ovariectomized animal served as a demonstrator and the intact animal as an observer. In S groups, the demonstrators were removed from their home cages and subjected to Pavlovian contextual fear conditioning, whereas observers remained in home cages in an isolated room. The training consisted of a 1 min adaptation period and 10 footshocks lasting 1 sec, of 1 mA intensity, which were applied with interstimulus intervals of 59 sec. The animals were removed from the experimental cage 1 min after the last footshock was applied. Immediately after training, the demonstrators were placed back into the home cage. In the NS groups the experimental procedure was identical, albeit no footshocks were applied to the demonstrators. Following 10 minutes of interaction, the animals were transported to the animal house. 24 hours following the training session, contextual fear was assessed in demonstrators by placing them in the conditioning chamber for 3 minutes and assessing their freezing levels.

### Experiment 1b: Analysis of pain thresholds in demonstrator rats

#### Subjects

Experiment 1b was performed one week after Experiment 1a using the same group of experimental subjects.

#### Apparatus

Pain thresholds were assessed using a LI7106 light beam analgesimeter (Letica). Thermal nociceptive stimulus was provided by a halogen light (12 V DC, 100 W) placed 35 mm above the groove, in which the animal’s tail was placed. The light beam was focused on the surface of animals’ tail. Stimulus intensity was set to 2 in an arbitrary scale of 1–10.

#### Procedure

The animal was restrained by the experimenter so that its tail was lying freely in the groove. Three measurements were performed, at 4, 5 and 6 cm from the base of the tail, and a mean tail flick latency was calculated. In order to habituate the animals to the restraint, two habituation sessions were performed on two consecutive days preceding the experiment.

### Experiment 2: Behavior of observers of different sex and, in females, estrus cycle phases, during an interaction with fear-conditioned demonstrators

#### Subjects

Subjects were 98 young adult Long-Evans rats, 34 males and 64 females, weighing approx. 250–300 g and 160–210 g at the beginning of the experiment, respectively. The animals were obtained and housed as described in Experiment 1a.

#### Estrus cycle phase assessment

Estrus cycle phase assessment was carried out daily in observer rats. Vaginal swabs were taken between 10:00 and 12:00 a.m. and assessed using a light microscope. Female observers in the estrus phase, characterized by the presence of numerous un-nucleated, cornified cells in the vaginal smear, as well as in the diestral phase, when the smear contained sparse amounts of un-nucleated and nucleated cells as well as leukocytes, were included in the experiment.

#### Procedure

Until the interaction of the observer with the demonstrator, the procedure was the same as in described in Experiment 1a. The interaction was videotaped and analyzed. Subsequently, the observers were subjected to two-way avoidance training (as described in Experiment 3). The video files were analyzed by a person blind to the experimental conditions using BehaView software (http://pmbogusz.net).

### Experiment 3: Two-way avoidance learning in observers of different sex and, in females, estrus cycle phases, following an interaction with a fear-conditioned demonstrator rat

#### Subjects

The same subjects (observers only; 17 males and 32 females), the behavior of which was analyzed in Experiment 2, were subsequently included in Experiment 3.

#### Behavioral apparatus

The training was carried out in a shuttle box 510 mm (W) ×250 mm (D) ×240 mm (H) (Panlab). The shuttle box was situated in a separate room. Side and rear walls were made of opaque black Plexiglas and the hinged front door was constructed from translucent Plexiglas. The ceiling was made of aluminium and had a Plexiglas easy-access doors above each compartment. The floor consisted of two separate grid floors (one for each compartment) constructed from 18 stainless steel rods (3.0 mm diameter). The floors were wired to a shocker/scrambler (Panlab) for delivery of footshocks (US). The central loudspeaker provided by the manufacturer was replaced by two loudspeakers placed on the side walls, 19 cm above the floor. A white noise generator was used as a conditioned stimulus (CS) source. The animal’s movement was detected by weight transducers (Panlab). The chamber was controlled and data was acquired using SHUTAVOID software (Panlab). Between tests, the chambers were cleaned with a detergent solution.

#### Procedure

Immediately following the interaction with the demonstrator, the observer was subjected to two-way avoidance training. The animal was placed in the left compartment of the shuttle box, facing the side wall. After 20 sec of adaptation period, the first trial started with 70 dB white noise (CS). 5 sec following the introduction of CS, a 0.8 mA footshock was introduced (US). Moving to the opposite compartment of the shuttle box within the first 5 sec of the CS prevented the introduction of US, automatically terminated the CS and was scored as an avoidance response. A analogous response that was initiated after the introduction of US automatically terminated both the CS and US and was scored as an escape response. The maximum duration of US was 30 sec. Intertrial intervals (ITI’s) lasted from 10 to 30 sec and were selected randomly. Crossings between compartments were allowed during adaptation periods and ITI’s, and were scored as intertrial responses (ITR’s). Latencies of instrumental responses as well as ITR’s were automatically recorded. Training session was terminated 20 sec after the last trial. A single training session consisted of 50 trials and 2 training sessions were performed on consecutive days.

### Experiment 4: c-Fos protein expression in limbic structures of demonstrator and observer rats

#### Subjects

Subjects were 96 young adult Long-Evans rats, 34 males and 64 females, weighing respectively approx. 250–300 g and 160–210 g at the beginning of the experiment. The animals were obtained and housed as described in Experiment 1a.

#### Procedure

To the point of interaction of the demonstrator with the observer, the procedure was the same as described in Experiment 1a. Following the interaction, the animals were transported to the animal house. 90 minutes after return of the demonstrator rat to the homecage, the animals were deeply anaesthetized with a mix of ketamine and xylazine (dosage as described in Experiment 1). Subsequently, the animals were perfused intracardially with ice-cold phosphate-buffered saline (PBS) followed by 4% paraformaldehyde solution in PBS. Their brains were removed and post-fixed in 4 % paraformaldehyde in PBS overnight at 4 °C. Following that, the brains were immersed in 30% sucrose solution in PBS for 5 days at 4 °C, frozen over dry ice and stored at –80 °C. For immunohistochemical stainings, brains were sectioned coronally at 40 μm in a cryotome. Sections containing prefrontal cortex (2.7 to 3.2 anterior to bregma), insular cortex (0.25 to 0.4 posterior to bregma) as well as central and basolateral amygdaloid nuclei (2.1 to 3.3 posterior to bregma) were collected. Immunohistochemcal stainings were performed as described previously^15^.

### Statistical analysis

The normality of data distribution within groups was assessed using the Shapiro-Wilk W test. When distribution was normal, factorial ANOVA and LSD test for post-hoc comparisons were used. When data distribution was significantly different from normal, non-parametric methods were used for further analysis. Significant differences between groups were identified with Kruskal-Wallis and Mann-Whitney tests. In case of cumulated latencies, data were compared using Kolomogorov-Smirnov two-sample test.

## Additional Information

**How to cite this article**: Mikosz, M. *et al.* Sex differences in social modulation of learning in rats. *Sci. Rep.* **5**, 18114; doi: 10.1038/srep18114 (2015).

## Figures and Tables

**Table 1:** Experimental groups.

Treatment		Male(M)	Estral Female (E)	Diestral Female (D)
Shocked (S)	Demonstrator (D)	MS-D	ES-D	DS-D
	Observer (O)	MS-O	ES-O	DS-O
Non-shocked (NS)	Demonstrator (D)	MNS-D	ENS-D	DNS-D
	Observer (O)	MNS-O	ENS-O	DNS-O

**Table 2:** Summary of mean numbers and durations of the behaviors observed during a 10-min social interaction (± SEM).

Behavior		MS-O	MNS-O	DS-O	DNS-O	ES-O	ENS-O
Rearing	time [s]	279.2 ± 21.1	307 ± 24.7^1^	228.1 ± 26.4	192.4 ± 29.4^1^	295.1 ± 22.4	256.9 ± 29.5
	number	84.1 ± 3.9	84.2 ± 6.1	81 ± 8.7	70.8 ± 10.3	89.88 ± 3	87.14 ± 7.8
Digging	time [s]	6.7 ± 2.6	6.8 ± 2.8	2 ± 1.4	1.9 ± 1.2	4.9 ± 2.9	5.4 ± 3.6
	number	3 ± 1.1	3.2 ± 1.2	1.2 ± 1	1.4 ± 0.8	2.3 ± 1.1	2.6 ± 2
Short grooming	time [s]	3.15 ± 1.7	8.59 ± 2.4	3.59 ± 2.2	9.85 ± 3.9	1.41 ± 0.9	2.92 ± 2
	number	1 ± 0.3	1.67 ± 0.4	0.5 ± 0.2	1.25 ± 0.5	0.38 ± 0.3	0.57 ± 0.3
Long grooming	time [s]	6.75 ± 3.5	0	6.1 ± 3.3	4 ± 3.3	8.3 ± 3.8	5.7 ± 5
	number	0.4 ± 0.2	0	1 ± 0.4	0.4 ± 0.2	0.6 ± 0.2	0.3 ± 0.2
Sniffing (head area)	time [s]	21.82 ± 6.5	22.04 ± 3.3^2^,^3^	20.34 ± 6.3	11.95 ± 3.4^2^	18.04 ± 5.3	6.62 ± 1.9^3^
	number	22.1 ± 4.2	27.89 ± 3.7^1^,^3^	20.33 ± 5.1	11.38 ± 3^1^	17.75 ± 4.4	9.86 ± 2^3^
Sniffing (anogenital area)	time [s]	28.71 ± 5.6	24.92 ± 4.8	19.15 ± 5	15.33 ± 2.6	24.01 ± 2.4	21.25 ± 5.2
	number	25 ± 3.7	23.11 ± 4	17.33 ± 3.7	15.25 ± 2.6	24.12 ± 2.6	20.14 ± 4.7
Allogrooming	time [s]	0	0.2 ± 0.2	0	0.7 ± 0.7	0.5 ± 0.5	1.2 ± 1
	number	0	0.1 ± 0.1	0	0.1 ± 0.1	0.1 ± 0.1	0.3 ± 0.2

^1^MNS-O>DNS-O, p < 0.01,

^2^MNS-O>DNS-O, p < 0.05,

^3^MNS-O>ENS-O, p < 0.01.
